# Optimized Microstructure and Improved Magnetic Properties of Pr-Dy-Al-Ga Diffused Sintered Nd-Fe-B Magnets

**DOI:** 10.3390/ma14102583

**Published:** 2021-05-16

**Authors:** Pengpeng Qu, Feifei Li, Sajjad Ur Rehman, Lei He, Xiaoqiang Yu, Qingfang Huang, Munan Yang, Jiajie Li

**Affiliations:** 1 Jiangxi Key Laboratory for Rare Earth Magnetic Materials and Devices, Institute for Rare Earth Magnetic Materials and Devices (IREMMD), Jiangxi University of Science and Technology, Ganzhou 341000, China; qppwrx@163.com (P.Q.); lifeifei1997@yeah.net (F.L.); sajjadurehman@gmail.com (S.U.R.); helyangy@163.com (L.H.); yuxiaoqiang@jxust.edu.cn (X.Y.); sduhqf@163.com (Q.H.); yangmunan@jxust.edu.cn (M.Y.); 2 College of Rare Earths, Jiangxi University of Science and Technology, Ganzhou 341000, China; 3 Fujian Rare-Earth Function Material Key Laboratory, Longyan 364000, China; 4 National Rare Earth Functional Materials Innovation Center, Ganzhou 341000, China

**Keywords:** Nd-Fe-B magnets, grain boundary diffusion process, magnetic properties, thermal stability, recoil loops

## Abstract

The grain boundary diffusion process (GBDP) has become an important technique in improving the coercivity and thermal stability of Dy-free sintered Nd-Fe-B magnets. The influence of Dy_70_Al_10_Ga_20_ and (Pr_75_Dy_25_)_70_Al_10_Ga_20_ alloys by the GBDP on sintered Nd-Fe-B magnets are investigated in this paper. After diffusing Dy_70_Al_10_Ga_20_ and (Pr_75_Dy_25_)_70_Al_10_Ga_20_ alloys, the coercivity (*H*_cj_) of the magnets increased from 13.58 kOe to 20.10 kOe and 18.11 kOe, respectively. Meanwhile, the remanence of the magnets decreased slightly. The thermal stability of the diffused magnets was improved by the GBDP. The microstructure shows continuous Rare-earth-rich (RE-rich) grain boundary phases and (Dy, Pr/Nd)_2_Fe_14_B core-shell structures which contribute to improving the coercivity. Moreover, the Dy concentration on the surface of the (Pr_75_Dy_25_)_70_Al_10_Ga_20_ diffused magnets decreased with the Pr substitution for the Dy element. The openness of the recoil loops for the (Pr_75_Dy_25_)_70_Al_10_Ga_20_ diffused magnets is smaller than that of the original magnets and Dy_70_Al_10_Ga_20_ diffused magnets. The results show that the (Pr_75_Dy_25_)_70_Al_10_Ga_20_ alloys can effectively optimize the microstructure and improve the magnetic properties and thermal stability of the sintered Nd-Fe-B magnets.

## 1. Introduction

Sintered Nd-Fe-B magnets possessing excellent high intrinsic coercivity and energy products are widely used in wind power, hybrid vehicles, maglev trains, and household appliances, etc. [1,2]. In this application, higher magnetism is required. Since the invention of Nd-Fe-B magnets in 1983, the remanence (*J*_r_) and the maximum energy product (*BH*)_max_ of the Nd-Fe-B magnets reached the theoretical values, while the *H*_cj_ is only 30% of the theoretical value [3]. However, the higher *H*_cj_ is urgently proposed in the face of increasingly harsh working environments, especially in high-temperature and high-humidity climates [4,5,6]. There are basically two ways to develop high coercivity. One is to improve the inherent temperature dependence of *H*_cj_, and the other is to develop higher coercivity at room temperature to resist thermal demagnetization of the magnets when exposed to high temperature. Heavy rare earth elements Dy/Tb can be substituted for Pr/Nd to increase the magneto-crystalline anisotropy field (*H*_A_), causing a substantial enhancement of *H*_cj_ by a single alloying method. However, due to the antiferromagnetic coupling between Dy and Fe, it is unfavorable to the saturation magnetization [7,8]. In order not to sacrifice the saturation magnetization, the GBDP (grain boundary diffusion process) technique was proposed by Park et al. [9]. Heavy rare earth elements can be selectively diffused into the magnet interior along the grain boundary (GB), forming a hard core-shell structure surrounding the main grains. Later, researchers successively used heavy rare earth metals/compounds/alloys containing Dy or Tb, such as Dy, Dy_2_O_3_, DyF_3_, DyH_2_, and Dy-Cu or Pr-Dy-Cu [10,11,12,13,14] acting as diffusion sources to improve the *H*_cj_.

In the GBDP, there are large amounts of the Dy/Tb element enriched on the magnets’ near-surface by diffusing the Dy/Tb, which causes thicker Dy/Tb-rich shells. Some findings show that the thickness of the Tb-rich shell can be reduced by controlling the thickness of TbF_3_ coatings and Al-aided TbH_2_ powders’ diffusion [15,16], which can still enhance the *H*_cj_ increment. Therefore, we can design suitable diffusion resources to decrease the thickness of the Dy/Tb-rich shell on the near-surface of the magnets.

In addition, the non-rare-earth elements Al/Ga/Cu can also increase the *H*_cj_ of the sintered Nd-Fe-B magnets and reduce the irreversible loss of magnetic flux. These elements mainly enrich in the RE-rich (Rare-earth rich) liquid phase to improve the wettability and increase the coercivity of the magnets [17,18,19]. Therefore, we select the ternary alloy Pr_70_Al_10_Ga_20_ and Dy_70_Al_10_Ga_20_ and quaternary alloy (Pr_75_Dy_25_)_70_Al_10_Ga_20_ as the diffusion sources in this work. The (Pr_75_Dy_25_)_70_Al_10_Ga_20_ alloys inculcate the best properties with a large substitution of Pr for Dy after the GBDP, as shown in Supplementary Material. The magnetic properties and thermal stability of the diffused magnets are analyzed. The relationship between microstructure and recoil loops and diffusion mechanism of the magnets are also discussed.

## 2. Materials and Methods

The commercial sintered Nd-Fe-B magnet of N52 was selected and wire-cut into small magnets with a size of φ10 × 10 × 5 mm^3^, and the chemical composition was (Pr, Nd)_30_Co_1.0_Cu_0.15_Zr_0.12_Ga_0.3_B_0.94_Fe_bal._ (wt.%). The ingots of Pr_70_Al_10_Ga_20_, Dy_70_Al_10_Ga_20_, and (Pr_75_Dy_25_)_70_Al_10_Ga_20_ (wt.%) were prepared by arc melting under a high-purity argon atmosphere. Then, these ingots were melt-spun into ribbons with 10 mm in width and 0.17 mm in thickness at a speed of 8 m/s. The magnets were polished with 400 mesh, 800 mesh, 1000 mesh, 1500 mesh, and 2000 mesh sandpaper and then ultrasonically washed in alcohol and dried. The ribbons were placed on the top and bottom of the magnet and put in a sintering furnace for diffusion heat treatment. The heat treatments were carried out at 850 °C for 6 h, and then annealed at 490 °C for 3 h in a vacuum (10^−4^ Pa) tubular furnace. The magnetic properties at different temperatures were measured by a boron hydride tracer (NIM-500C, National Institute of Metrology, Beijing, China). The melting points of the alloys were measured by differential scanning calorimetry (DSC250, TA Instruments, USA). Additionally, the microstructure of the magnets was observed by a field emission scanning electron microscope (FESEM, MLA650F, FLIR Systems, Inc., Wilsonville, OR, USA). The irreversible magnetic flux loss at elevated temperatures was measured by pulling Helmholtz coils. The phase constitution of the magnets was determined by the X-ray diffraction with a Cu-K_α_ radiation (XRD, D8 Advance, Bruker, Billerica, MA, USA). The elemental distribution of Nd-Fe-B magnets was explored by using an electron probe microanalyzer (EPMA, JXA-8530F, JEOL, Tokyo, Japan). The recoil loops of the magnets were measured by the Physical Property Measurement System (PPMS-DynaCOOL1-9, Quantum Design, San Diego, CA, USA) in fields up to 5 T at room temperature.

## 3. Results and Discussion

**Figure 1:**
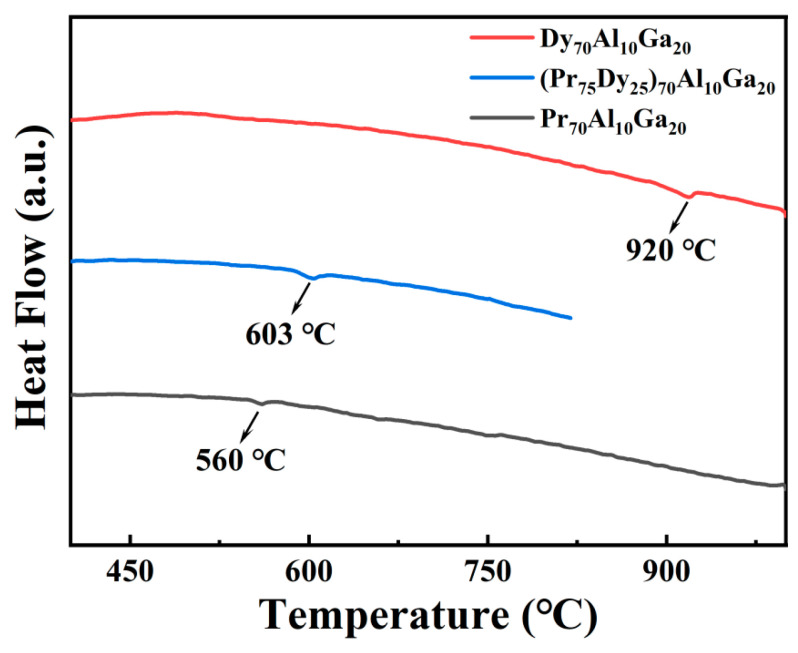
DSC results of Dy_70_Al_10_Ga_20_, (Pr_75_Dy_25_)_70_Al_10_Ga_20_, and Pr_70_Al_10_Ga_20_ ribbons.

Figure 1 shows the DSC results of the Dy_70_Al_10_Ga_20_, (Pr_75_Dy_25_)_70_Al_10_Ga_20_, and Pr_70_Al_10_Ga_20_ ribbons. Compared with ternary alloys, quaternary alloys have relatively lower melting points. Additionally, the lower melting point may reduce the activation energy of the diffusion and improve diffusion efficiency. Moreover, compared with the ternary alloy Dy_70_Al_10_Ga_20_, the quaternary alloy (Pr_75_Dy_25_)_70_Al_10_Ga_20_ contains less heavy rare earth elements; thus, it reduces the costs.

**Figure 2:**
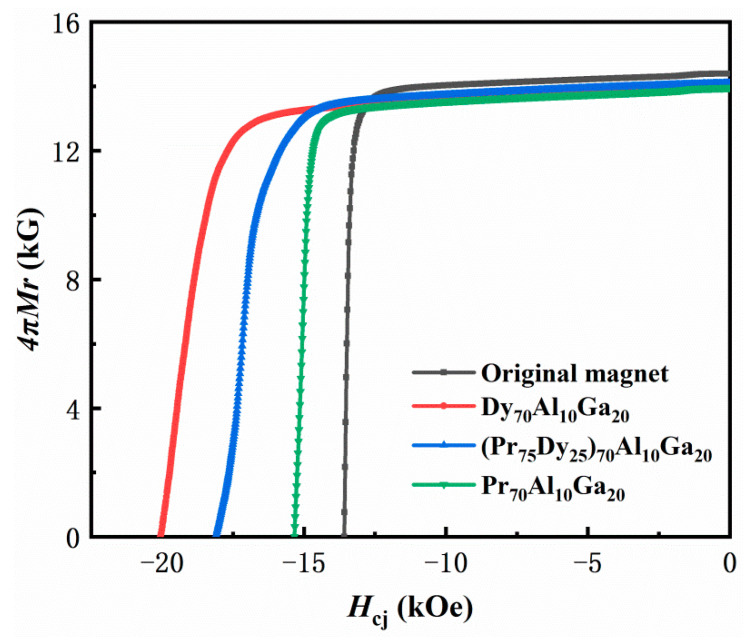
Demagnetization curves of the original magnet and Pr_70_Al_10_Ga_20_, Dy_10_Al_10_Ga_20_ and (Pr_75_Dy_25_)_70_Al_10_Ga_20_ diffused magnets.

Figure 2 shows demagnetization curves at the room temperature of the original magnets and Pr_70_Al_10_Ga_20_, Dy_70_Al_10_Ga_20_, and (Pr_75_Dy_25_)_70_Al_10_Ga_20_ diffused magnets. It can be clearly seen that the *H*_cj_ of the diffused magnets is improved after the GBDP, while the remanence is only slightly decreased. The coercivity increased from 13.58 kOe to 15.34 kOe, 20.10 kOe, and 18.11 kOe, respectively, after diffusing Pr_70_Al_10_Ga_20_, Dy_70_Al_10_Ga_20_, and (Pr_75_Dy_25_)_70_Al_10_Ga_20_ alloys. At the same time, the *J*_r_ reduced from 14.3 kG to 14.0 kG, 14.0 kG, and 14.1 kG, respectively. The increase in the *H*_cj_ is mainly because of the partial substitution of Pr and Dy for Nd to form the core-shell structure of (Dy, Pr/Nd)_2_Fe_14_B. The antiferromagnetic coupling of Dy and Fe atoms reduces the *J*_r_ of Dy_70_Al_10_Ga_20_ and (Pr_75_Dy_25_)_70_Al_10_Ga_20_ diffused magnets. In addition, the Pr content in the Pr_70_Al_10_Ga_20_ diffused magnet is higher, and the nonmagnetic volume fraction increases after diffusion, which leads to the decrease in the *J*_r_. The increase in the volume fraction of the non-magnetic phases in the grain boundary is another reason for the decrease in the *J*_r_.

**Figure 3:**
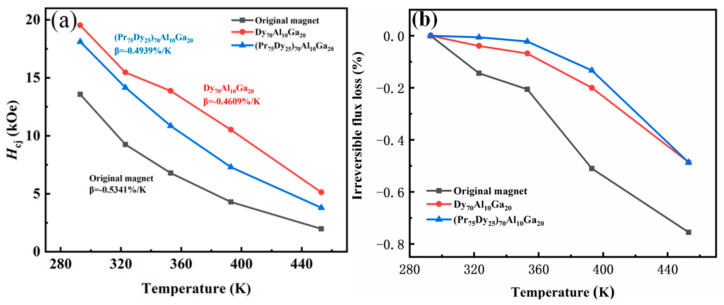
(**a**) the coercivity curves of the original magnets and the Dy_70_Al_10_Ga_20_ and (Pr_75_Dy_25_)_70_Al_10_Ga_20_ diffusion magnets in the temperature range of 293–453 K; (**b**) the irreversible flux loss curve of the original magnets and the diffused Dy_70_Al_10_Ga_20_ and (Pr_75_Dy_25_)_70_Al_10_Ga_20_ magnets at 293–453 K.

Figure 3a shows the coercivity curves of the original magnet and the Dy_70_Al_10_Ga_20_ and (Pr_75_Dy_25_)_70_Al_10_Ga_20_ diffused magnets at the temperature range of 293 to 453 K. Additionally, the temperature coefficient of coercivity (*β)* of the magnets can be calculated according to the formula [20,21]:

*βH*_cj_(*T*_0_) = [*H*_cj_(*T*_1_) − *H*_cj_(*T*_0_)]/(*T*_1_ − *T*_0_) × 100% (1)

where *T*_1_ is the elevated temperature, and *T*_0_ is the room temperature. The *β* increased from −0.5341 %/K of the original magnets to −0.4609 %/K and −0.4939 %/K, respectively, for the Dy_70_Al_10_Ga_20_ and (Pr_75_Dy_25_)_70_Al_10_Ga_20_ diffused magnets. Figure 3b is the irreversible flux loss curve of the original magnet and the diffused Dy_70_Al_10_Ga_20_ and (Pr_75_Dy_25_)_70_Al_10_Ga_20_ magnets at 293–453 K. The irreversible flux loss rates of the original magnet and the Dy_70_Al_10_Ga_20_ and (Pr_75_Dy_25_)_70_Al_10_Ga_20_ alloy diffused magnets were 75.5%, 48.6%, and 48.7%, respectively. The irreversible flux loss of the magnets was reduced by about 27% after diffusion, which suggests that the diffused magnets have less magnetic irreversible flux losses. The magnetic flux and coercivity are very sensitive to temperature. The structure loss occurs at a high temperature, which leads to demagnetization. The irreversible flux loss is not recoverable when back to room temperature. It is related to the irreversible change of the microstructure of the magnets. The GB microstructure of the diffused magnets was optimized after thermal diffusion treatment. The nucleation of the reverse magnetic domain of the magnets is suppressed, and it is difficult to trigger the magnetization reversal of the magnetic domain due to the hardening of the epitaxial layer of the matrix phase grain [22]. This results in an improvement in temperature coefficients and irreversible magnetic flux losses of the diffused magnets. These results indicate that the thermal stability of the diffused magnets was improved after the GBDP.

**Figure 4:**
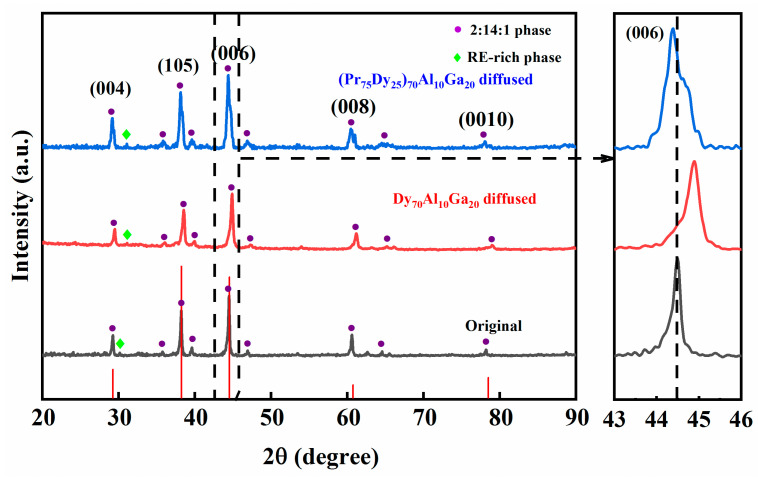
XRD patterns of the original magnet, and Dy_70_Al_10_Ga_20_ and (Pr_75_Dy_25_)_70_Al_10_Ga_20_ alloy diffused magnets (the surface of observation is the near-surface).

Figure 4 shows the XRD of the original magnets and the Dy_70_Al_10_Ga_20_ and (Pr_75_Dy_25_)_70_Al_10_Ga_20_ diffused magnets (vertical to the c-axis plane, with the observation surface near the surface). As can be seen from the diffraction peaks marked in Figure 4, most of the diffraction peaks are the main phases, and small parts are the RE-rich phase, and no new diffraction peaks appear in the diffused magnets. The characteristic diffraction peaks were located at 29.3, 44.6, 60.8, and 78.5 of 2θ, and Bragg diffraction peaks corresponding to (00l) are compared with JCPDS (Joint Committee on Powder Diffraction Standards) card no. 39-0473. This indicates that the magnets are dominated by 2:14:1 phases before and after the GBDP, and the content of other impurity phases is relatively small. The partially enlarged view of the diffraction peaks of the (006) crystal plane shows that the main phase peak of the magnet shifted to a large angle direction after diffusing Dy_70_Al_10_Ga_20_, while the main phase peak moved slightly to a small angle direction after diffusing (Pr_75_Dy_25_)_70_Al_10_Ga_20_. This is because the atomic radius of Dy (0.1773 nm) is smaller than Nd (0.1821 nm). According to the Bragg equation, when the Dy atoms diffuse into the main phase to replace Nd to form the (Nd, Dy)_2_Fe_14_B shell layer, the lattice parameters decrease. For the (Pr_75_Dy_25_)_70_Al_10_Ga_20_ diffused magnet, the lattice parameters of the main phase increase, which is because the diffusion amount of Pr is greater than that of Dy. Based on the Lanthanide contraction effect, the atomic radius of the Dy element is smaller than that of Pr and Nd. Dy instead of Pr/Nd makes the diffraction peaks move to the large angle; in contrast, Pr and Nd move the peak to a small angle. Consequently, the combined effect is that the diffraction peak shifts to a small angle. The shift of the peak also means that Pr and Dy have entered into the main phase, forming a stronger *H*_A_ of (Dy, Pr/Nd)_2_Fe_14_B shells, thus exhibiting the coercivity enhancement effect.

**Figure 5:**
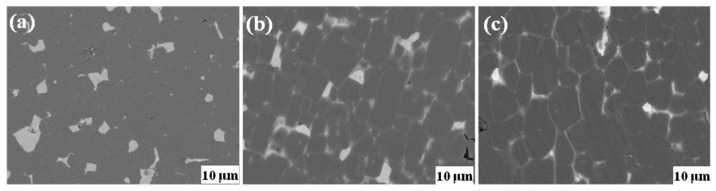
(**a–c**) are BSE-SEM images of the original magnet, and the Dy_70_Al_10_Ga_20_ and (Pr_75_Dy_25_)_70_Al_10_Ga_20_ alloy diffused magnets (the surface of observation is the near-surface), respectively.

In order to explore the reason for the *H*_cj_ enhancement, the microstructure of the magnets was observed after the GBDP. Figure 5a–c are BSE-SEM (backscattered electron) images of the original magnets, and the Dy_70_Al_10_Ga_20_ and (Pr_75_Dy_25_)_70_Al_10_Ga_20_ alloy diffused magnets, respectively. The dark gray parts in Figure 5 correspond to the 2:14:1 matrix phase grains, and the bright white and gray white areas correspond to the RE-rich phases. The bright white and gray white in SEM are caused by the difference in composition of the RE-rich phases. Figure 5a shows that the triple junction RE-rich phases of the original magnets were distributed discretely in the magnet interior, and some adjacent matrix phase grains were in direct contact, which is unfavorable to the *H*_cj_. Comparably, the smooth and continuous thin grain boundary RE-rich phases were formed in the Dy_70_Al_10_Ga_20_ and (Pr_75_Dy_25_)_70_Al_10_Ga_20_ diffused magnets. If all grains are surrounded by thin grain boundary phases, then the grains are magnetically isolated from each other. If the grains are in direct contact with each other, there will be a localized exchange coupling effect, and, as a result, the grains are connected together to form a larger ferromagnetic domain grain group. A small grain inversion will drive demagnetization of adjacent grains in chains, because there is no thin layer RE-rich phase boundary, which will not hinder the displacement of the domain wall [23]. Demagnetization of one grain will drive demagnetization of other grains, thus reducing coercivity; that is, the demagnetization resistance will be reduced.

**Figure 6:**
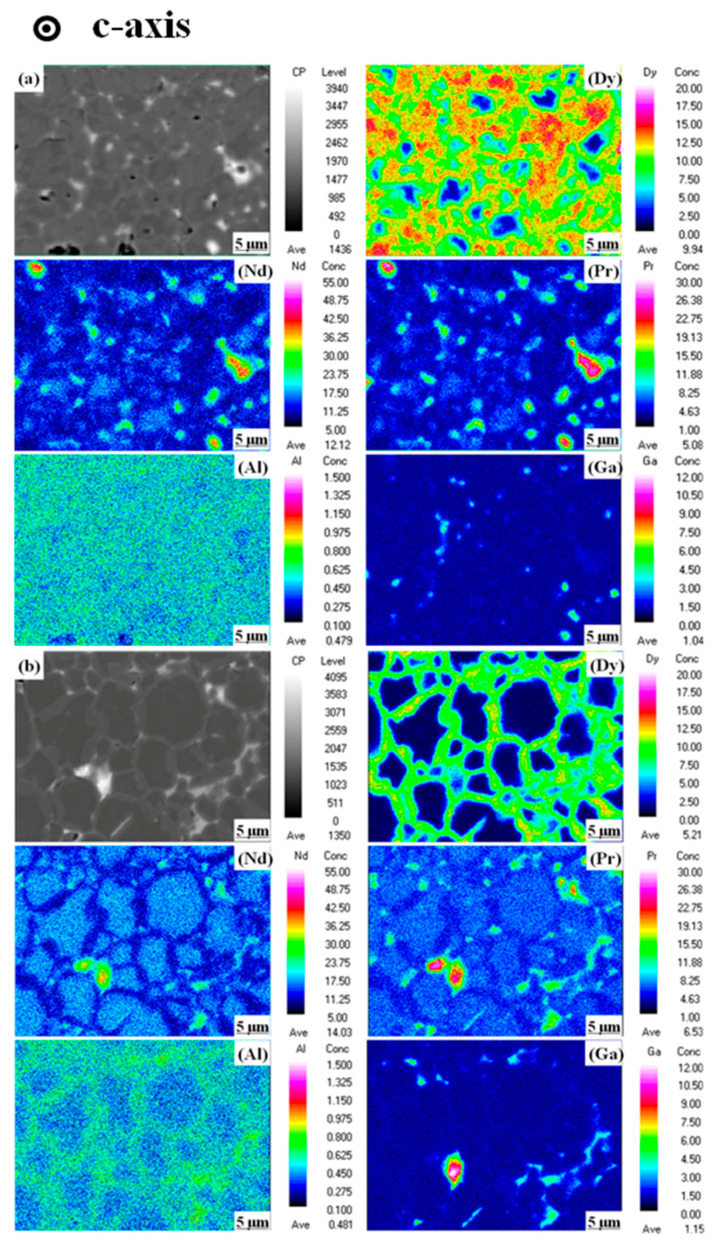
The EPMA mappings of the near-surface layer (perpendicular to c-axis): (**a**) Dy_70_Al_10_Ga_20_ magnet; (**b**) (Pr_75_Dy_25_)_70_Al_10_Ga_20_ magnet.

Figure 6 shows the EPMA images on the surface (perpendicular to c-axis) of the Dy_70_Al_10_Ga_20_ and (Pr_75_Dy_25_)_70_Al_10_Ga_20_ diffused magnets. The distribution of Dy, Nd, Pr, Al, and Ga elements in the Dy_70_Al_10_Ga_20_ and (Pr_75_Dy_25_)_70_Al_10_Ga_20_ magnets are shown in Figure 6 after the GBDP, respectively. Dy elements are mainly distributed in the main phase grain epitaxial layer to form the (Dy, Pr/Nd)_2_Fe_14_B core-shell structure, which is beneficial to increase the coercivity. During the GBDP, Dy penetrates into the Nd-Fe-B sintered magnets through liquid grain boundaries. The Dy-rich shells are only selectively formed on the low-index lattice plane of the main phase grains. These planes, generated by the partial melting of the main phase grains, offer the low-energy configurations at the Nd_2_Fe_14_B/GB interfaces [24]. During the subsequent cooling process, the Dy-rich liquid phases precipitate on the edge of the main phase grains and solidify to form (Nd, Dy)_2_Fe_14_B hard shells. As shown in Figure 6a, a large amount of Dy elements accumulated on the surface of the Dy_70_Al_10_Ga_20_ diffused magnets, while the enrichment on the (Pr_75_Dy_25_)_70_Al_10_Ga_20_ diffused magnets is mitigated. Although the *H*_cj_ of the (Pr_75_Dy_25_)_70_Al_10_Ga_20_ diffused magnet is 2 kOe lower than that of the Dy_70_Al_10_Ga_20_ diffused one, the heavy rare earth content of the quaternary alloy is much lower than the ternary alloy.

The grain boundary channels, and intergranular regions of the sintered Nd-Fe-B magnets, are typically around 100–1000 nm in size, and the high temperature wettability causes enough capillary thrust for these elements to enter the intergranular channels during liquefaction, which in turn causes the uniform distribution of grain boundaries with the matrix grains [25,26]. According to the distribution of Al and Ga in Figure 6b, most of them remain in the grain boundaries and play a role in wetting the grain boundaries [18]. At the same time, a small amount of Al also exists in the matrix grains, which is possible when the surface of the Nd-Fe-B grains is partially decomposed, and Dy replaces Nd atoms. Meanwhile, Al penetrates into the selected grain facets from the grain boundaries with a high concentration at the grain edges. With a cooling effect coming in place, lighter Al atoms get transported inwards due to the low melting point while matrix restructuring happens, known as core-shell morphology [27]. Although the core-shells are not obvious, a higher concentration of Pr at the grain boundaries takes precedence of surface diffusion by the substitution of Nd atoms, resulting in the intergranular region becoming richer with Nd and hard phase grains taking composition (Pr, Nd)_2_Fe_14_B. Therefore, under the combined effect of the above elements, the hard core-shell structure and optimized microstructure can explain the reason why the diffused magnets have an increased *H*_cj_ after GBDP.

**Figure 7:**
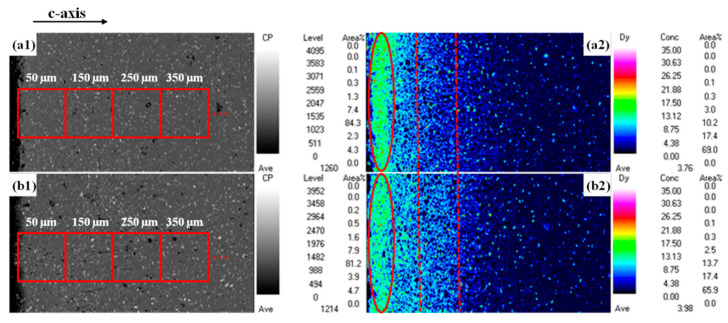
The SEM images and the corresponding EPMA mapping (parallel to c-axis) at 0–400 μm of (**a1**,**a2**) Dy_70_Al_10_Ga_20_ and (**b1**,**b2**) (Pr_75_Dy_25_)_70_Al_10_Ga_20_ diffused magnets, respectively.

To investigate the diffusion depth of Dy in different diffused magnets, the EPMA was performed to determine the distribution of the Dy element along the diffusion direction. Figure 7(a1,a2,b1,b2) show the corresponding EPMA mappings at 0–400 μm of Dy_70_Al_10_Ga_20_ and (Pr_75_Dy_25_)_70_Al_10_Ga_20_ diffused magnets. As can be seen from Figure 7(a2,b2), a high concentration of the Dy-rich area is formed on the surface of the magnet, and the Dy-rich area is indicated by the red ellipses in Figure 7(a2,b2). It can be seen from the red rectangular box that the concentration of the Dy element in the (Pr_75_Dy_25_)_70_Al_10_Ga_20_ diffused magnets is higher than that of the Dy_70_Al_10_Ga_20_ diffused magnets. With the diffusion depth increasing, the Dy-rich area gradually decreases. At the depth of 400 μm, the Dy element still exists in the magnet interior. At the same time, it is observed that the concentration of Dy in the (Pr_75_Dy_25_)_70_Al_10_Ga_20_ diffused magnets is higher than that of the Dy_70_Al_10_Ga_20_ diffused magnets at the same depth as the dotted lines in Figure 7(a2,b2). Therefore, the quaternary alloy (Pr_75_Dy_25_)_70_Al_10_Ga_20_ can save the Dy elements and promote its diffusion depth.

**Figure 8:**
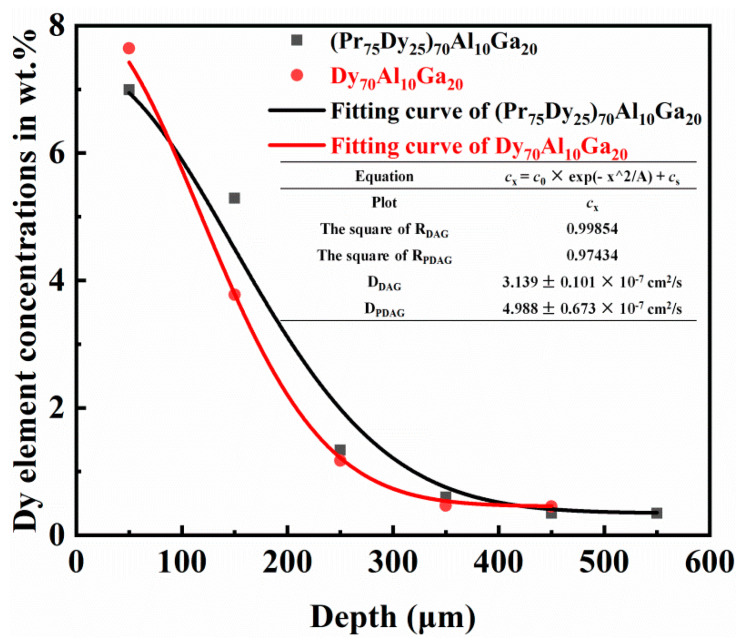
The fitting curves of Dy element concentration in (Pr_75_Dy_25_)_70_Al_10_Ga_20_ diffused magnets at different depths.

For sintered Nd-Fe-B, the GB provides a channel for the diffusion source. The melting point of the RE-rich grain boundary phase is about 655 °C, which is much lower than the melting point of the main phase of 1185 °C [28]. The element diffusion follows the Fick’s second law, which states that in the process of unsteady diffusion, we get

(2) $$c_{x}=c_{0}\times \exp(-x^{2}/A)+c_{s}$$

where *c_x_*, *c*_0_, and *c*_s_ are the volume concentrations of the diffusion material (kg/m^3^) at the different depths; *A* is a fixed value (when the surface concentration and time are determined); and *x* is the distance (m) [7]. Figure 8 shows the fitting curve of the Dy element concentration in the range of different depths in the diffused magnets. Additionally, the diffusion coefficients of the Dy element are approximately 4.988 ± 0.673 × 10^−7^ cm^2^/s and 3.139 ± 0.101 × 10^−7^ cm^2^/s in the (Pr_75_Dy_25_)_70_Al_10_Ga_20_ and Dy_70_Al_10_Ga_20_ diffused magnets, respectively. This also shows that the diffusion efficiency of the Dy elements in the quaternary alloys (Pr_75_Dy_25_)_70_Al_10_Ga_20_ is improved under the cooperation of the Pr elements. The concentration of the Dy element can be measured by EPMA along the diffusion direction from the 0 μm to 450 μm in a continuous 100 × 100 μm^2^ square indicated by the red boxes in Figure 7(a1,b1). As the diffusion depth increases, the concentration of Dy elements decreases, and the diffusion rate slows down.

**Figure 9:**
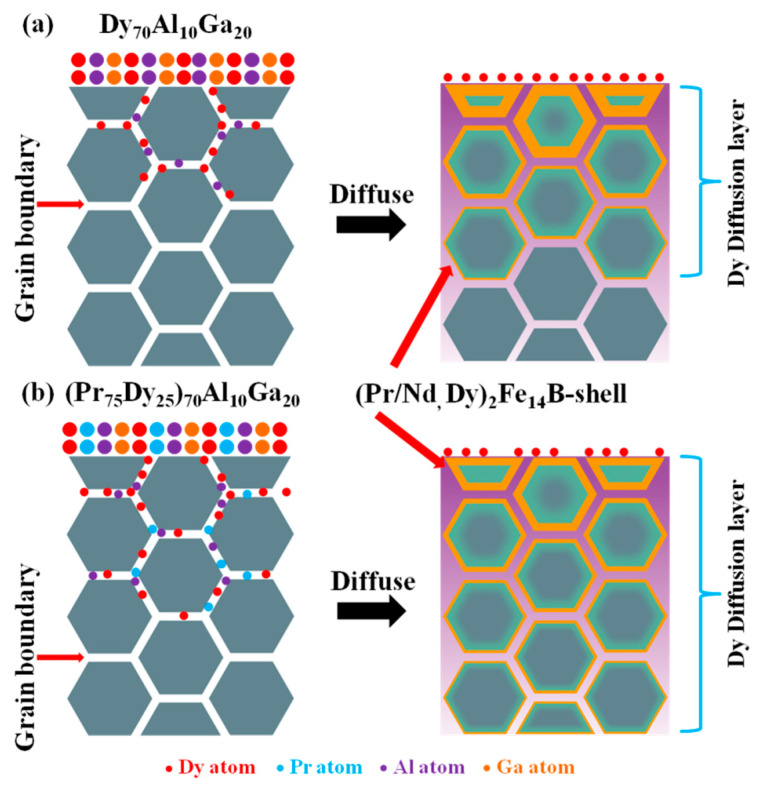
Schematic diagram of the Nd-Fe-B magnet diffused structure. (**a**) the Dy_70_Al_10_Ga_20_ diffused magnet; (**b**) the (Pr_75_Dy_25_)_70_Al_10_Ga_20_ diffused magnet.

Figure 9 gives a schematic diagram of the change in the amount of diffusion distinguished from the depth of the diffused magnets [29]. Figure 9a,b show the diffusion mechanism of ternary alloys Dy_70_Al_10_Ga_20_ and quaternary alloys (Pr_75_Dy_25_)_70_Al_10_Ga_20_, respectively. During the heat treatment, Dy atoms enter the magnet along the grain boundaries. By replacing Nd atoms with Dy atoms, a thin layer with higher Dy concentration is formed on the edge of the main phase grains, which is called the core-shell structure. The quaternary alloys (Pr_75_Dy_25_)_70_Al_10_Ga_20_ have the coordinated diffusion of the Pr element, so that the Dy element can penetrate deeper into the magnets and form a more core-shell structure. At the same time, the surface Dy concentration of the magnets can be regulated by diffusing (Pr_75_Dy_25_)_70_Al_10_Ga_20_ alloys. Due to the magnetic isolation effect of the grain boundaries and the high magnetocrystalline anisotropy field of the core-shell structure, the coercivities of the diffused magnets show improvement after the GBDP treatment.

**Figure 10:**
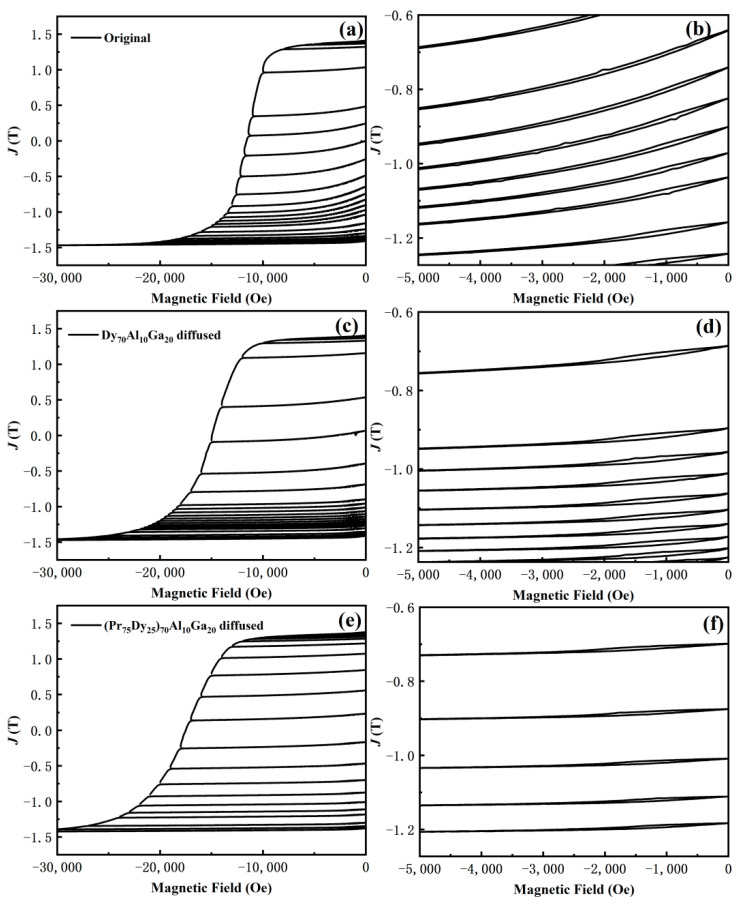
Recoil loops for (**a**,**b**) original magnets, and (**c**,**d**) Dy_70_Al_10_Ga_20_ and (**e**,**f**) (Pr_75_Dy_25_)_70_Al_10_Ga_20_ alloy diffused magnets.

The recoil loops can verify the magnitude of the demagnetization capability and the uniformity of the microstructure [17]. Figure 10 shows the recoil loops of the original magnets, and the Dy_70_Al_10_Ga_20_ and (Pr_75_Dy_25_)_70_Al_10_Ga_20_ diffused magnets. It shows that the recoil loops’ opening of the original magnet is larger, while that of the Dy_70_Al_10_Ga_20_ and (Pr_75_Dy_25_)_70_Al_10_Ga_20_ diffused magnets are much smaller. This is because the distribution of the RE-rich phase for the original magnet is non-uniform and discontinuous, and the grain boundary of the Dy_70_Al_10_Ga_20_ and (Pr_75_Dy_25_)_70_Al_10_Ga_20_ diffused magnets are optimized to be more uniform and continuous after the GBDP. However, a large amount of the Dy element enrichment on the surface of the Dy_70_Al_10_Ga_20_ diffused magnets leads to the larger opening of the recoil loops than that of the (Pr_75_Dy_25_)_70_Al_10_Ga_20_ diffused magnets. The reduced surface Dy enrichment improves the microstructure uniformity by diffusing the (Pr_75_Dy_25_)_70_Al_10_Ga_20_ alloy; thus, the recoil loops’ opening of the (Pr_75_Dy_25_)_70_Al_10_Ga_20_ diffused magnets is smaller than that of the original and Dy_70_Al_10_Ga_20_ diffused magnets. This is also confirmed by the microstructure of the magnets mentioned in Figure 5 and Figure 6.

## 4. Conclusions

In this paper, the effects of diffusing Dy_70_Al_10_Ga_20_ ternary alloys and (Pr_75_Dy_25_)_70_Al_10_Ga_20_ quaternary alloys on the magnetic properties and microstructure of sintered Nd-Fe-B magnets were investigated.

(1) The coercivity of the Pr_70_Al_10_Ga_20_, Dy_70_Al_10_Ga_20_ and (Pr_75_Dy_25_)_70_Al_10_Ga_20_ alloys diffused Nd-Fe-B magnets increased from 13.58 kOe to 15.34 kOe and 20.10 kOe and 18.11 kOe, respectively, while the remanence is only slightly decreased.
(2) The thermal stability of the diffused magnets improves by diffusing Dy_70_Al_10_Ga_20_ and (Pr_75_Dy_25_)_70_Al_10_Ga_20_ alloys. The *β* increased from −0.5341 %/K for the original magnets to −0.4609 %/K and −0.4939 %/K for the Dy_70_Al_10_Ga_20_ and (Pr_75_Dy_25_)_70_Al_10_Ga_20_ diffused magnets, respectively.
(3) The optimized GB microstructure and (Dy, Pr/Nd)_2_Fe_14_B core-shell structure hardening around the main grains isolate the 2:14:1 phases, which are the main reasons for the great improvement of coercivity in the diffused magnets.
(4) The decreased surface Dy enrichment and the optimized grain boundary microstructure lead to the smaller opening of the recoil loops by diffusing the (Pr_75_Dy_25_)_70_Al_10_Ga_20_ alloy. This indicates that the diffused magnet has a stronger capability for demagnetization.

## Data Availability

Data sharing is not applicable to this article.

## Supplementary Materials

The following are available online at https://www.mdpi.com/article/10.3390/ma14102583/s1, Figure S1: Demagnetization curve of the (Pr_100-x_Dy_x_)_70_Al_10_Ga_20_ (x = 0, 25, 50, 75, 100) diffused magnets.
