# Supplementary material for: Optimized Microstructure and Improved Magnetic Properties of Pr-Dy-Al-Ga Diffused Sintered Nd-Fe-B Magnets
Source: Materials (Basel). 2021 May 16;14(10):2583. doi: 10.3390/ma14102583 (PMC8156186; doi:10.3390/ma14102583)
Supplement: Supplementary file 1 [file materials-14-02583-s001.zip › materials-1203792-supplementary.pdf]

materials

Supplementary Materials

Optimized Microstructure and Improved Magnetic Properties of Pr-Dy-Al-Ga Diffused Sintered Nd-Fe-B Magnets

Pengpeng Qu [1,2], Feifei Li [1,2], Sajjad Ur Rehman [1,2], Lei He [1,2], Xiaoqiang Yu [1,2], Qingfang Huang [1,3], Munan Yang [1,2,4] and Jiajie Li [1,2,4,*]

1 Jiangxi Key Laboratory for Rare Earth Magnetic Materials and Devices, Institute for Rare Earth Magnetic Materials and Devices (IREMMD), Jiangxi University of Science and Technology, Ganzhou 341000, China; qppwrx@163.com (P.Q.); lifeifei1997@yeah.net (F.L.); sajjadurehman@gmail.com (S.U.R.); helyangy@163.com (L.H.); yuxiaoqiang@jxust.edu.cn (X.Y.); sduhqf@163.com (Q.H.); yangmunan@jxust.edu.cn (M.Y.)
2 College of Rare Earths, Jiangxi University of Science and Technology, Ganzhou 341000, China
3 Fujian Rare-earth Function Material Key Laboratory, Longyan 364000, China
4 Nation Rare Earth Functional Materials Innovation Center, Ganzhou 341000, China
* Correspondence: lijiajie@jxust.edu.cn

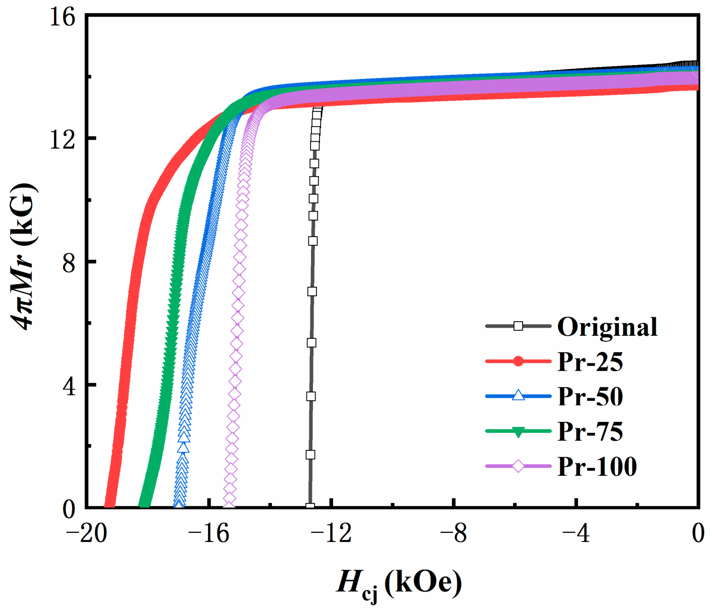

Figure S1. Demagnetization curve of the $(Pr_{100-x}Dy_x)_{70}Al_{10}Ga_{20}$ (x = 0, 25, 50, 75, 100) diffused magnets.
